# Chitosan oligomers (COS) trigger a coordinated biochemical response of lemongrass (*Cymbopogon flexuosus*) plants to palliate salinity-induced oxidative stress

**DOI:** 10.1038/s41598-023-35931-w

**Published:** 2023-05-27

**Authors:** Mohammad Mukarram, M. Masroor A. Khan, Daniel Kurjak, Francisco J. Corpas

**Affiliations:** 1 Advance Plant Physiology Section, Department of Botany, Aligarh Muslim University, Aligarh, 202002 India; 2 Department of Phytology, Faculty of Forestry, Technical University in Zvolen, T. G. Masaryka 24, 96001 Zvolen, Slovakia; 3 Department of Integrated Forest and Landscape Protection, Faculty of Forestry, Technical University in Zvolen, T. G. Masaryka 24, 96001 Zvolen, Slovakia; 4 Department of Stress, Development and Signaling in Plants, Group of Antioxidant, Free Radical and Nitric Oxide in Biotechnology, Food and Agriculture, Estación Experimental del Zaidín, Consejo Superior de Investigaciones Científicas (CSIC), Granada, Spain

**Keywords:** Plant physiology, Plant stress responses

## Abstract

Plant susceptibility to salt depends on several factors from its genetic makeup to modifiable physiological and biochemical status. We used lemongrass (*Cymbopogon flexuosus*) plants as a relevant medicinal and aromatic cash crop to assess the potential benefits of chitosan oligomers (COS) on plant growth and essential oil productivity during salinity stress (160 and 240 mM NaCl). Five foliar sprays of 120 mg L^−1^ of COS were applied weekly. Several aspects of photosynthesis, gas exchange, cellular defence, and essential oil productivity of lemongrass were traced. The obtained data indicated that 120 mg L^−1^ COS alleviated photosynthetic constraints and raised the enzymatic antioxidant defence including superoxide dismutase (SOD), catalase (CAT), and peroxidase (POD) activities that minimised salt-induced oxidative damage. Further, stomatal conductance (g_s_) and photosynthetic CO_2_ assimilation (*A*) were improved to support overall plant development. The same treatment increased geraniol dehydrogenase (GeDH) activity and lemongrass essential oil production. COS-induced salt resilience suggests that COS could become a useful biotechnological tool in reclaiming saline soil for improved crop productivity, especially when such soil is unfit for leading food crops. Considering its additional economic value in the essential oil industry, we propose COS-treated lemongrass as an excellent alternative crop for saline lands.

## Introduction

A common denominator during salt stress is the overproduction of reactive oxygen species (ROS)^1,2^. ROS, though capable of metabolic signalling during optimal environment, oxidises biological macromolecules (proteins, lipids, DNA) in abundance^3,4^. The aftereffect of which can include growth and productivity retardation or cellular death in plants. The survival of plants under such a scenario relies on the integration of stress and adaptive physiological and anatomical changes^5,6^. A group of counter-oxidative compounds plays a pivotal part in shielding ROS-induced damage. These compounds, antioxidants, are majorly localised in chloroplast, mitochondria, and peroxisomes, which are also the primary ROS-producing sites^7–11^. Osmolytes are a different set of compounds responsible for maintaining osmotic homeostasis during stress conditions^12,13^. Both the antioxidants [e.g., superoxide dismutase (SOD), catalase (CAT), peroxidase (POD)] and osmolytes [e.g., proline (PRO)] altogether influence the extent of oxidative damage and counter-response of plants to salt stress^14,15^.

Various ‘new-age’ growth elicitors along with augmenting growth, development, and yield of plants, galvanise plant defence system against environmental stressors including salinity^16–18^. The benefit of incorporating such elicitors in agricultural practices lies in their ecological superiority over traditional ones. Among these elicitors, chitosan has attracted many biologists and agricultural scientists for its biocompatibility, biodegradability, bioactivity, non-toxicity, ubiquity, and inexpensiveness^19,20^. Chitosan is a linear polymer of randomly distributed β-(1,4)-2-amino-2-deoxy-d-glucose (deacetylated unit) and *N*-acetyl-d-glucosamine (acetylated unit) and is commercially obtained from the alkaline deacetylation of chitin^21–23^. Many industries such as pharmaceuticals and food safety and preservation, incorporate chitosan and its derivatives for their sought-after functionalities^24–27^. In agriculture, chitosan improves plant growth, development, productivity, and stress tolerance through defensive gene activation^16,28–30^. The breaking down of chitosan polymers into oligomeric subunits through irradiation or digestion of its β-1,4-glycosidic bonds between monomeric sugar residues through partial acidic, alkaline, or enzymatic action further upgrades its structural and functional properties^31–33^. The lower molecular weight and smaller size of chitosan oligomers or chitooligosaccharides (COS) give them higher solubility, surface area, and fluidity, and they exhibit higher efficacy of desired effects over their polymeric counterparts^34–36^. Independent studies have established COS conferred enhancement of many crops including *Oryza sativa*^37^, *Triticum aestivum*^38^, *Zea mays*^39^, *Hordeum vulgare*^40^, *Glycine max*^41^, *Coffea canephora*^42^, *Vitis vinifera*^43^, *Cymbopogon flexuosus*^44^, and *Phaseolus vulgaris*^45^ under both normal and stress conditions.

Thus, the working hypothesis for this study was that COS improve growth and production in the lemongrass and protect the plant during salt stress (H1). We further tested the hypothesis that the basis of this tolerance is the elicitation of ROS and antioxidant metabolism that corresponds to cellular homeostasis in lemongrass (H2). Lemongrass is a C_4_ perennial aromatic grass and is cultivated for its essential oil. Although both lemongrass^46^ and its essential oil^47^ have been known for a long time in human history, it was only recently when lemongrass essential oil (LEO) found extensive usage in medicinal, food safety and packaging, and cosmetic industries owing to its antimicrobial, antioxidant, anticancer, and insect-repellent activities^48,49^. During the past two decades, LEO export has risen by > 1250% in India, suggesting a substantial potential economic incentive from lemongrass cultivation (reviewed by Mukarram et al.^50^). This has encouraged contemporary researchers to use interactive approaches for enhancing lemongrass production^44,51–57^. Considering the exponentially growing lemongrass market and the massive economic loss due to salinity, it is a matter of interest to know if we can grow lemongrass crops in high salt-affected lands for their essential oil. The study, sensu lato, can be used in reclaiming saline lands with lemongrass cultivation converting such lands from economic liability to economic asset.

To test H2, we pinpointed a few cardinal components of the cellular defence system in lemongrass comprising the activity of the antioxidant enzymes SOD, CAT, and POD, and PRO content an osmoprotectant, for their activity trend against H_2_O_2_ (hydrogen peroxide) and TBARS (thiobarbituric acid reactive substances) accumulation under two different growing conditions of NaCl (160 and 240 mM). Given physiological adjustments during stressful environments are swifter than transcriptional changes, the metabolic analyses provide new insights into our understanding of the physiological response of lemongrass to salinity.

## Materials and methods

### Plant material and growth conditions

The slips of lemongrass [*Cymbopogon flexuosus* (Nees ex Steudel) Watson] var. Nima were purchased from the Central Institute of Medicinal and Aromatic Plants, Lucknow (India), as plant material for this study. After surface sterilisation with 0.2% HgCl_2_ for 5 min, slips were washed repetitively with deionised water. The plant slips were moved to a semi-controlled net-house at the Department of Botany, Aligarh Muslim University (AMU), Aligarh (27° 52′ N, 78° 51′ E, and 187 m a.s.l.) and 7 L capacity earthen pots (25 cm × 25 cm) filled with sand, clay, and peat (70:20:10 w/w). During evaluation time, maximum and minimum values for temperature were recorded at 36 °C and 27 °C (± 4 °C), respectively, while relative humidity was (74 ± 7%). Random soil collection from different pots was analysed at Soil-Testing Laboratory, Indian Agricultural Research Institute (IARI), New Delhi, and quantified as: texture-sandy loam, pH (1:2): 7.6, electrical conductivity (E.C.) (1:2): 0.52 m mhos cm^−1^, available nitrogen (N), phosphorus (P) and potassium (K): 94.8, 8.9, and 136.5 mg kg^−1^ of soil, respectively. All methods were carried out in accordance with relevant guidelines.

### COS preparation and structural analysis

Marine hydrocolloids in Kerala (India) provided un-irradiated chitosan. The chitosan was subjected to γ-irradiation at Bhabha Atomic Research Centre in Mumbai (India) utilising a Cobalt-60 source at 2.4 Kilo Gray (kGy) per hour dosage (250 kGy in total). A solvent of 1% acetic acid to prepare COS solution. The University Sophisticated Instrumentation Facility Centre at AMU in Aligarh, India employed scanning electron microscopy (JOEL, JSM-6510 LV, Japan) to conduct structural analysis on both chitosan and COS. This analysis verified that COS exhibited a smaller size compared to bulk chitosan.

### Induction of salt stress

*Cymbopogon flexuosus* slips were grown under two distinct NaCl concentrations: 160 mM and 240 mM. These salt levels were considered severe (160 mM) and extreme (240 mM) due to lemongrass's moderate salt sensitivity^58^. The salt treatments commenced 21 days after transplantation (21 DAT). To reach the desired concentration without causing osmotic shock, 300 mL of 40 mM NaCl solutions were applied every alternate day. In contrast, the control group received 300 mL of double distilled water exclusively.

### Chitosan treatments

**Figure 1:**
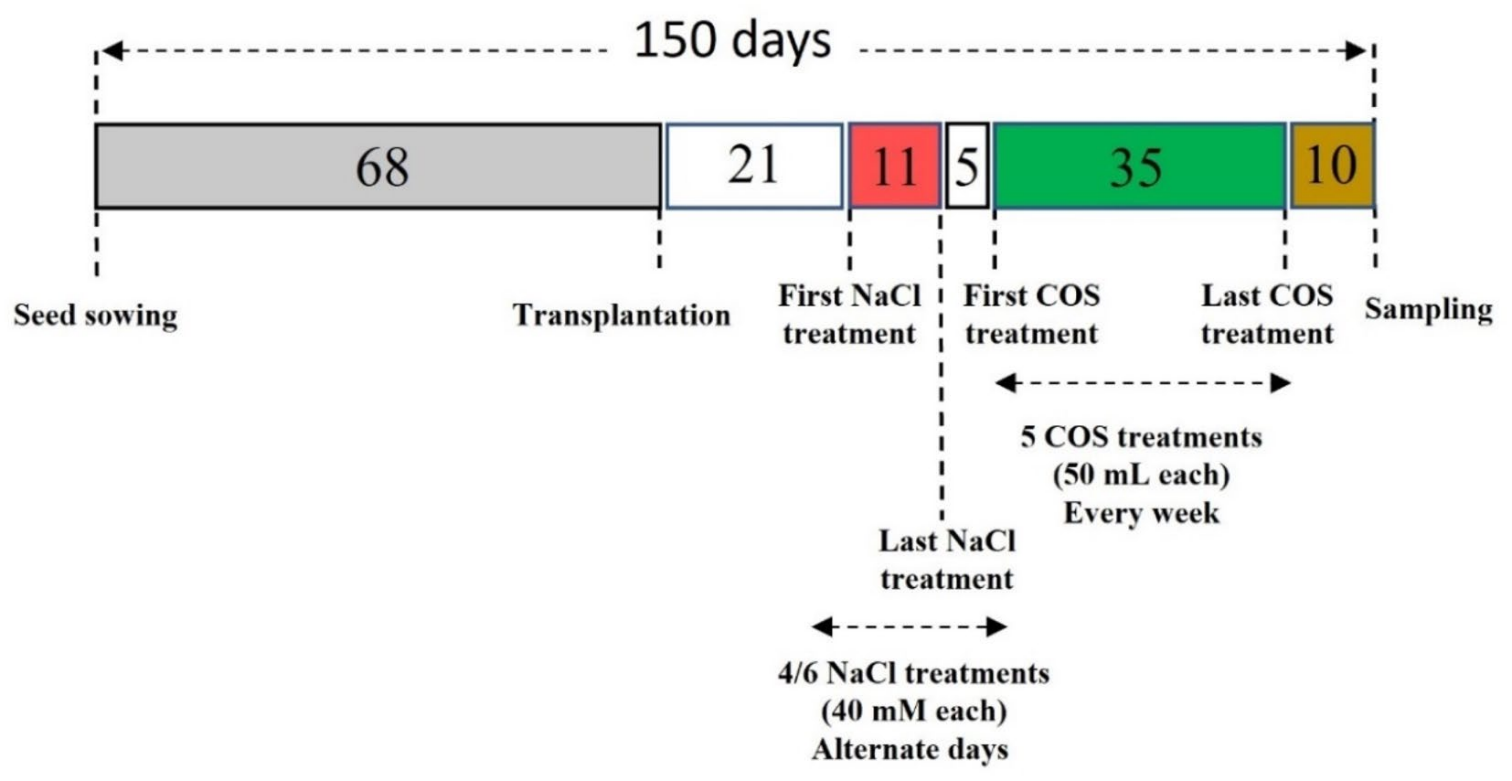
The experimental timeline of major events during the present study.

COS treatments were applied as foliar sprays using a hand sprayer. Based on our earlier findings, 120 mg L^−1^ of COS was given to the lemongrass plants^44^. In total, five foliar sprays (50 mL each) were applied every week starting 5 days after the attainment of the final salt concentration for each group. The schedule in Fig. 1 shows the experimental design used for NaCl and chitosan treatments.

### Photosynthesis and stomatal behaviour

To assess chlorophyll fluorescence (Fv/Fm), a saturation-pulse fluorometer PAM-2000 (Walz, Effeltrich, Germany) was utilised. The plants underwent a 30-min period of darkness to ensure dark adaptation before assessing photosynthetic efficiency. The adaxial surface of the first fully developed leaf was selected to note Fv/Fm during the daytime. The chlorophyll content in the intact extended leaves was quantified using a Minolta chlorophyll meter (SPAD-502; Konica Minolta Sensing Inc., Japan). For the assessment of photosynthetic carbon assimilation (*A*), stomatal conductance (*gs*), and transpiration rate (*E*) in the youngest fully expanded plant leaves, a portable Infra-red Gas Analyzer (LiCOR 6200, Portable Photosynthesis System, NA, USA) was employed. Before appraising *A*, *gs*, and *E*, a 2-min pre-acclimation of the leaves in the leaf cuvette head was conducted. All measurements were performed on 6 cm^2^ leaf block while retaining specific environmental conditions: air temperature at 25 °C, relative humidity between 65 and 85%, and atmospheric CO_2_ concentration at 370 ± 5 μmol mol^–1^. All assessments were conducted between 09:00 and 12:00 h when the photosynthetic photon flux density (PPFD) ranged from 780 to 800 μmol m^−2^ s^−1^.

### Quantification of oxidative burst

The H_2_O_2_ quantification was carried out using a peroxidase-dependent assay, following the method developed by Okuda et al.^59^. The reaction was started with peroxidase at room temperature (25 °C) and absorbance hike at 590 nm was monitored with a spectrophotometer for 3 min. The H_2_O_2_ was quantified as μmol H_2_O_2_ g^−1^ fresh weight (FW).

The TBARS amount was ascertained in the fresh leaf tissues by Cakmak and Horst^60^. TBARS were appraised in terms of malondialdehyde (MDA) equivalents (i.e., as nmol MDA g^−1^ FW). In summary, 0.5 g sample of fresh leaf tissues was finely ground with 5 mL of trichloroacetic acid solution (0.1% w/v). The resulting mixture was subjected to centrifugation at 12,000×*g* (5 min). Then, 1 mL supernatant aliquot was combined with 4 mL of tetrabutylammonium solution (0.5% w/v) in trichloroacetic acid (20% w/v). The mixture was incubated (30 min, 90 °C) and then put in an ice bath. After another round of centrifugation (10,000×*g*, 5 min), the supernatant’s optical density was spectrophotometrically quantified (Shimadzu UV-1700, Tokyo, Japan) at a wavelength of 532 nm. To account for any non-specific turbidity, the absorbance at 600 nm was subtracted from the obtained values.

### Preparation of leaf extracts

For the enzymatic assays, 0.2 g of fresh lemongrass leaves were ground in liquid N_2_ using a mortar and pestle. The resulting coarse powder (0.5 g) was transferred to 5 mL (w/v) of chilled extraction medium containing potassium phosphate buffer (100 mM, pH 7.8), 1% (w/v) polyvinylpyrrolidone and 0.5% (v/v) Triton-X-100. Homogenates were centrifuged at 15,000×*g* for 5 min at 4 °C. The supernatant acquired after centrifugation was used for the determination of enzymatic antioxidant activities^61^.

### Enzyme activity assays

The method of Beyer and Fridovich^62^ was used to determine the SOD activity (E.C. 1.15.1.1). Freshly formulated enzyme extract (0.1 mL) was mixed with riboflavin (1 mM), methionine (9.9 mM), NBT (nitrobluetetrazolium 55 mM), EDTA (2 mM), and Triton-X-100 (0.02%). The mixture was illuminated and maintained for one h at 30 °C, followed by spectrophotometric analyses (560 nm). SOD activity was expressed in SOD units. The amount of the SOD needed for half inhibition of the NBT reaction at the set wavelength is calculated as one unit.

The CAT activity (E.C. 1.11.1.6) was determined with the methods of Beers and Sizer^63^ with slight modification. 0.04 mL of the leaf extract was added to 2.6 mL of potassium phosphate buffer (50 mM with pH 7). The solution was centrifuged afterwards at 12,500×*g* for 20 min at 4 °C. The aliquot of the supernatant was removed, followed by substrate addition (0.4 mL of 15 mM H_2_O_2_) to the remaining solution. The enzyme activity of CAT was measured by determining the disappearance of H_2_O_2_ at 240 nm for 2 min with 5 s intervals.

The POD activity (EC 1.11.1.7) was measured by determining the amount of purpurogallin formed at 420 nm by adopting the methodology of Kumar and Khan^64^.

Leaf extract for geraniol dehydrogenase (GeDH, EC 1.1.1.183) activity was prepared by homogenising leaves into Tricine-NaOH (50 mM, pH 7.5), β-mercaptoethanol (2.5 mM), thiourea (5 mM), phenylmethylsulfonylfluoride (1 mM), and glycerol (15%, v/v) in the presence of Polyclar AT and amberlite XAD-4 as described in our earlier experiment^44^. Enzyme activity was assayed by determining geraniol-dependent-NADP^+^ reduction and recording absorbance increment at 340 nm. All enzymatic activity of antioxidants was expressed according to protein content in the samples.

The protein content in lemongrass leaf samples was done following Bradford's method^65^ using the bovine serum albumin to make the standard curve.

### Proline content

The estimation of proline content was conducted following the procedures outlined by Bates et al.^66^. Fresh leaves weighing 0.25 g were finely ground with sulfosalicylic acid (2.5 mL, 3%). After centrifuging the solution (10,000×*g*, 10 min), 2 mL supernatant aliquot was poured to a separate test tube with sulfosalicylic acid (2.5 mL), glacial acetic acid (1 mL), and acid ninhydrin solution (1 mL) followed by boiling (100 °C, 1 h) in a hot water bath. Then, an ice bath was used to stop the reaction. The extraction was performed by toluene (3 mL) followed by vigorous shaking of the mixture for 20–25 s. The solution was allowed to settle, separating the aqueous portion from the toluene-aspired layer. The toluene layer containing the chromophore was then measured spectrophotometrically for optical density at 520 nm.

### Evaluation of growth and productivity variables

Growth parameters were evaluated in terms of plant height, dry weight, and leaf area. For dry weights, plants were dried for 24 h at 80 °C in a hot-air oven. The leaf area was determined by the millimeter graph paper method^67^. The leaf was spread over the millimeter graph paper, and the leaf outline was marked. Afterwards, the marked area on the graph paper was cut and weighed (x). Additionally, 1 cm^2^ of the same paper was cut and weighed separately (y). The ratio of x/y depicted the leaf area (cm^2^).

Lemongrass oil was extracted by hydro-distillation of the leaves^68^. Lemongrass leaves (100 g) were cut into tiny portions and transferred to a flask associated with Clevenger’s apparatus (Borosil, India). Double-distilled water was added to this flask. Subsequently, the flask was heated over the heating mantle for 3 h. The vapour formed consisted of the essential oil mixed with water. The essential oil was collected into the receiver after passing through the condenser to cool.

### Statistical analysis

The normal distribution of the data was first tested for each treatment by the Shapiro–Wilk test. Barlett’s test assessed the homogeneity of variance among treated plants. The influence of chitosan on lemongrass morpho-physiology was tested through analysis of variance (one-way). Moreover, significant differences among treated plants were assessed through Duncan’s multiple range post-hoc test. All statistical analyses were conducted at the replicate level (n = 5) and α = 0.05 in SPSS-25.0 for Windows (SPSS, Inc., Chicago, IL, USA). Principal component analysis (PCA) was performed on the observed parameters using FactoMineR and factoextra packages to distinguish each treatment’s position. Additionally, all the variables were connected by the PerformanceAnalytics package and presented in the correlation matrix. Correlation analysis was used to analyse relationships among all parameters observed for control and treated plants.

## Results

### COS appease salinity-induced growth constraints in lemongrass

**Figure 2:**
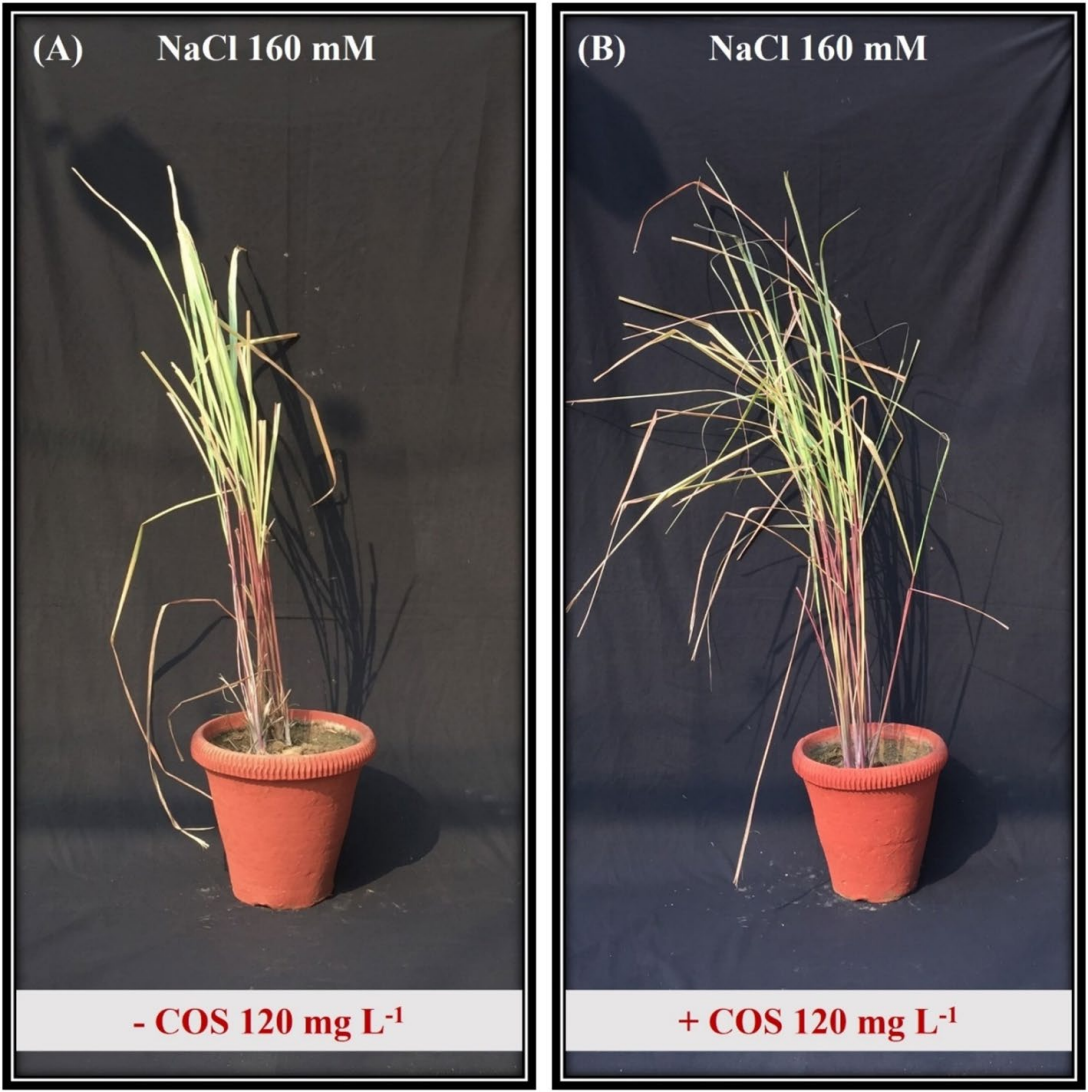
Phenotype of lemongrass plant under NaCl 160 mM salinity regime without (**A**) and with (**B**) COS application (120 mg L^−1^).

**Figure 3:**
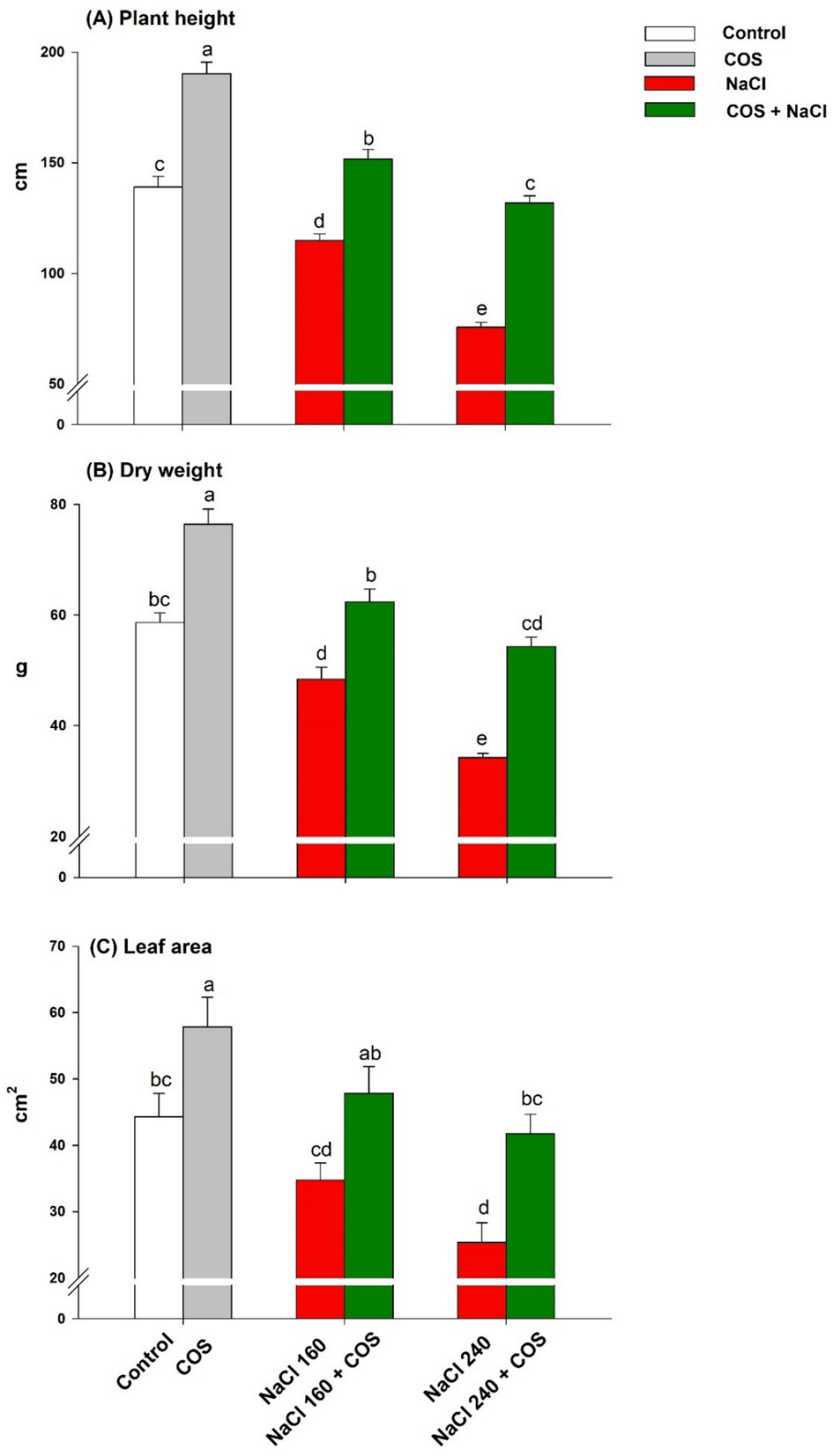
Effect of 120 mg L^−1^ chitosan oligomers (COS) on lemongrass plant height (**A**), dry weight (**B**), and leaf area (**C**) under salt stress. Five replicates mean ± standard error is represented for each bar. The difference between the mean values having the same letter(s) is insignificant (p ≤ 0.05) by the LSD test at a 5% probability level (α = 0.05). COS = 120 mg L^−1^. NaCl concentrations are represented in mM.

The visible effect of salt stress comprised redundant growth, shorter plants, and fewer green leaves (Fig. 2). The salt stress reduced plant height, dry weight, and leaf area under both NaCl concentrations (160 and 240 mM) over control (Fig. 3). The height and weight reduction were maximised in plants grown under NaCl 240 mM regime. However, when COS (120 mg L^−1^) was sprayed on these plants, plant height was improved by 37% (Fig. 3A). At the same time, leaf area was boosted by 31% (Fig. 3C). Similar COS superiority was observed in dry weight measurements where it completely reversed the salt effect during NaCl 160 mM (Fig. 3B).

### COS reverse salt-conferred effects on lemongrass photosynthesis and stomatal dynamics

**Figure 4:**
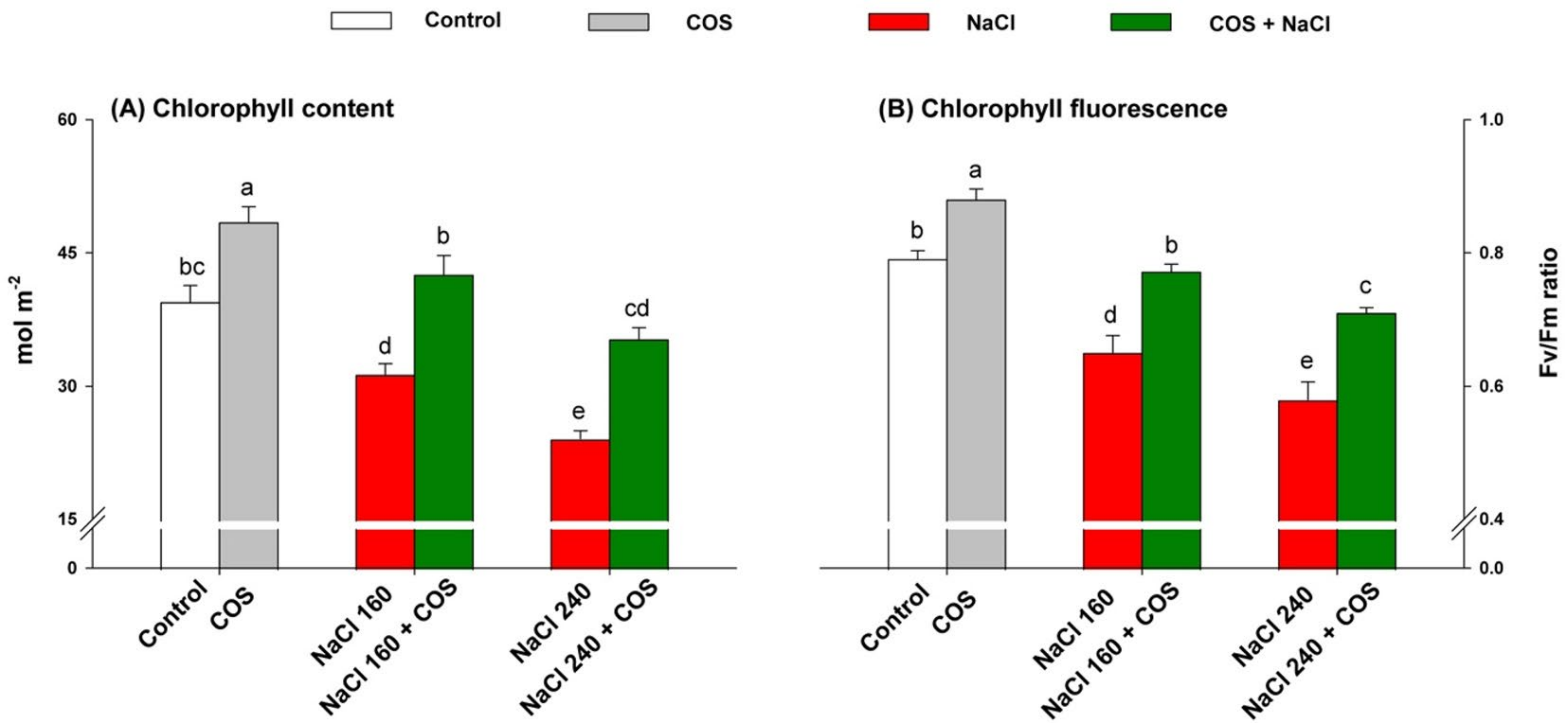
120 mg L^−1^ chitosan oligomers (COS) effect on chlorophyll content (**A**) and chlorophyll fluorescence (Fv/Fm) (**B**) of lemongrass under salinity. Five replicates mean ± standard error is represented for each bar. The difference between the mean values having the same letter(s) is insignificant (p ≤ 0.05) by the LSD test at a 5% probability level (α = 0.05). NaCl concentrations are represented in mM.

Lemongrass photosynthetic traits were determined in terms of chlorophyll content and Fv/Fm. All parameters exhibited more significant damage with increasing salt concentration. Therefore, the minimised photosynthetic activities were detected in lemongrass leaves raised under NaCl 240 mM. Nevertheless, spraying such leaves with COS 120 mg L^−1^ improved chlorophyll content (Fig. 4A) and Fv/Fm (Fig. 4B).

**Figure 5:**
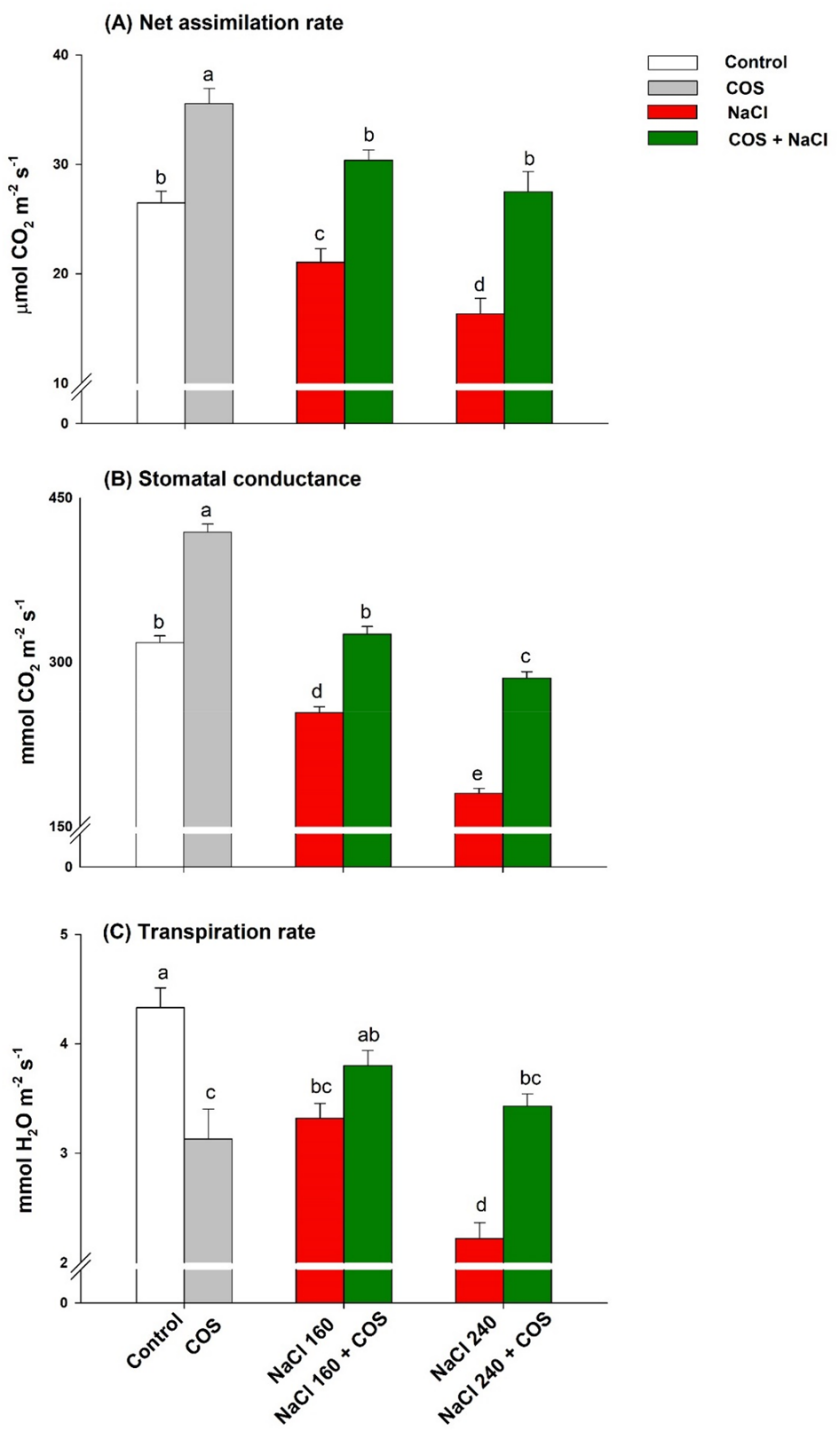
Influence of 120 mg L^−1^ of chitosan oligomers (COS) sprays on stomatal dynamics such as net CO_2_ assimilation rate (**A**), stomatal conductance (**B**), and transpiration rate (**C**) of lemongrass under salinity. Five replicates mean ± standard error is represented for each bar. The difference between the mean values having the same letter(s) is insignificant (p ≤ 0.05) by the LSD test at a 5% probability level (α = 0.05). NaCl concentrations are represented in mM.

Stomatal behaviour was severely restricted during saline settings regarding g_s_, *A*, and *E* (Fig. 5A–C). The NaCl 240 mM corresponded to the maximised reduction in g_s_ (Fig. 5A) and *A* (Fig. 5B) in the lemongrass leaves. Nevertheless, COS spray ameliorated saline constraints on g_s_ by 28% and 58% and on *A* by 44% and 68% in plants treated with NaCl 160 and 240 mM, respectively, over their stressed equivalents.

### COS upgrade redox metabolism during salinity

**Figure 6:**
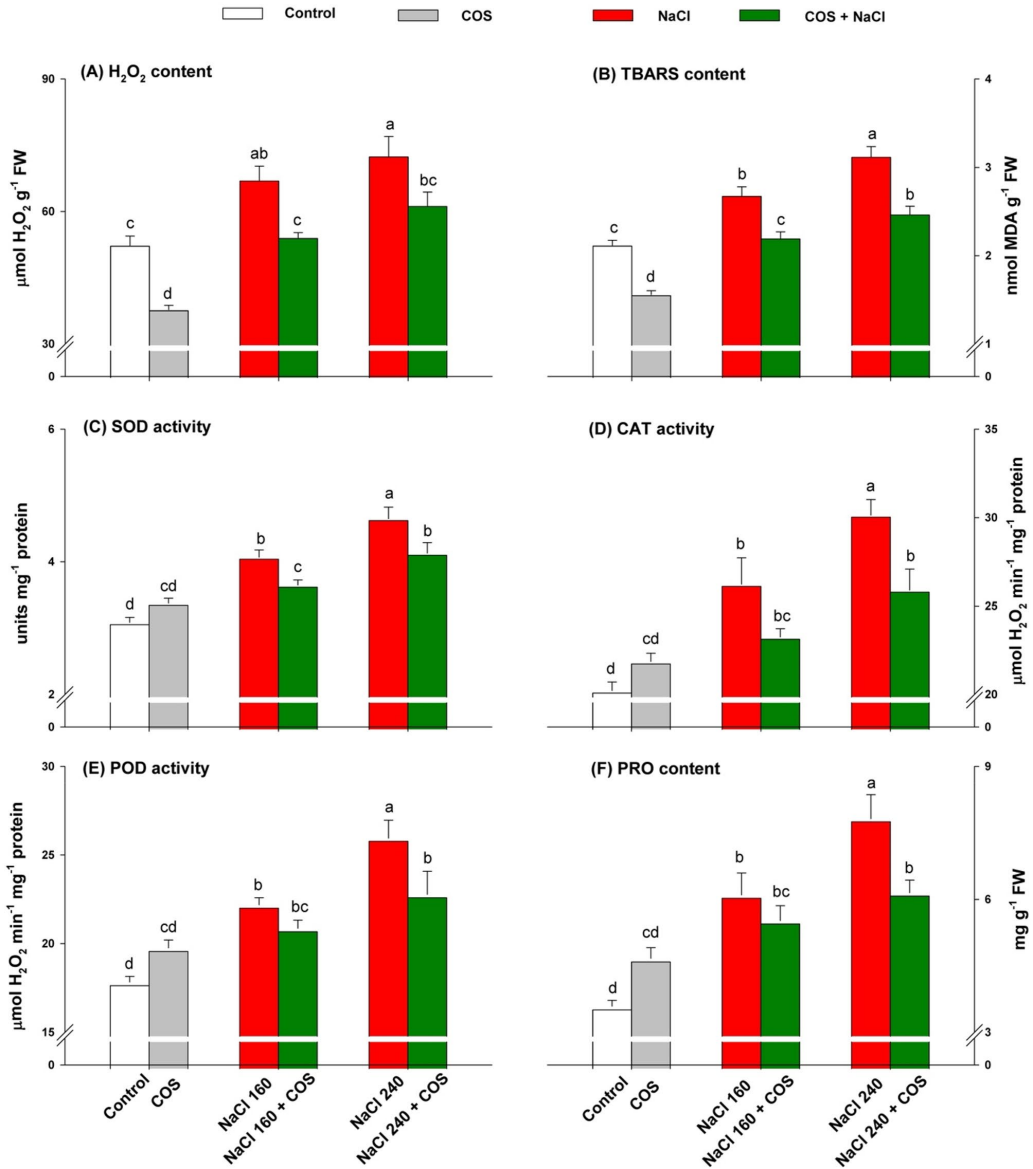
120 mg L^−1^ COS-induced antioxidative defence response in lemongrass during salt stress. Five replicates mean ± standard error is represented for each bar. The difference between the mean values having the same letter(s) is insignificant (p ≤ 0.05) by the LSD test at a 5% probability level (α = 0.05). NaCl concentrations are represented in mM. *H*_*2*_*O*_*2*_ hydrogen peroxide, *TBARS* thiobarbituric acid reactive substances, *SOD* superoxide dismutase, *CAT* catalase, *POD* peroxidase, *PRO* proline.

The H_2_O_2_ and TBARS contents were increased under both NaCl concentrations (160 and 240 mM), implying more significant oxidative damage (Fig. 6A,B). Nevertheless, COS diminished the H_2_O_2_ and TBARS contents in stressed plants. The highest antioxidant activities (SOD, CAT, and POD) were detected in plants treated with NaCl 240 mM (Fig. 6C–E). PRO content followed a similar trend (Fig. 6F). The smaller H_2_O_2_ and TBARS amounts required lesser antioxidative activities, demonstrated by decreased CAT, POD, and SOD activities when COS were sprayed on salt-stressed lemongrass individuals. A similar pattern was observed in PRO content with the COS treatments during both saline regimes.

### COS repair crop productivity under salt stress

**Figure 7:**
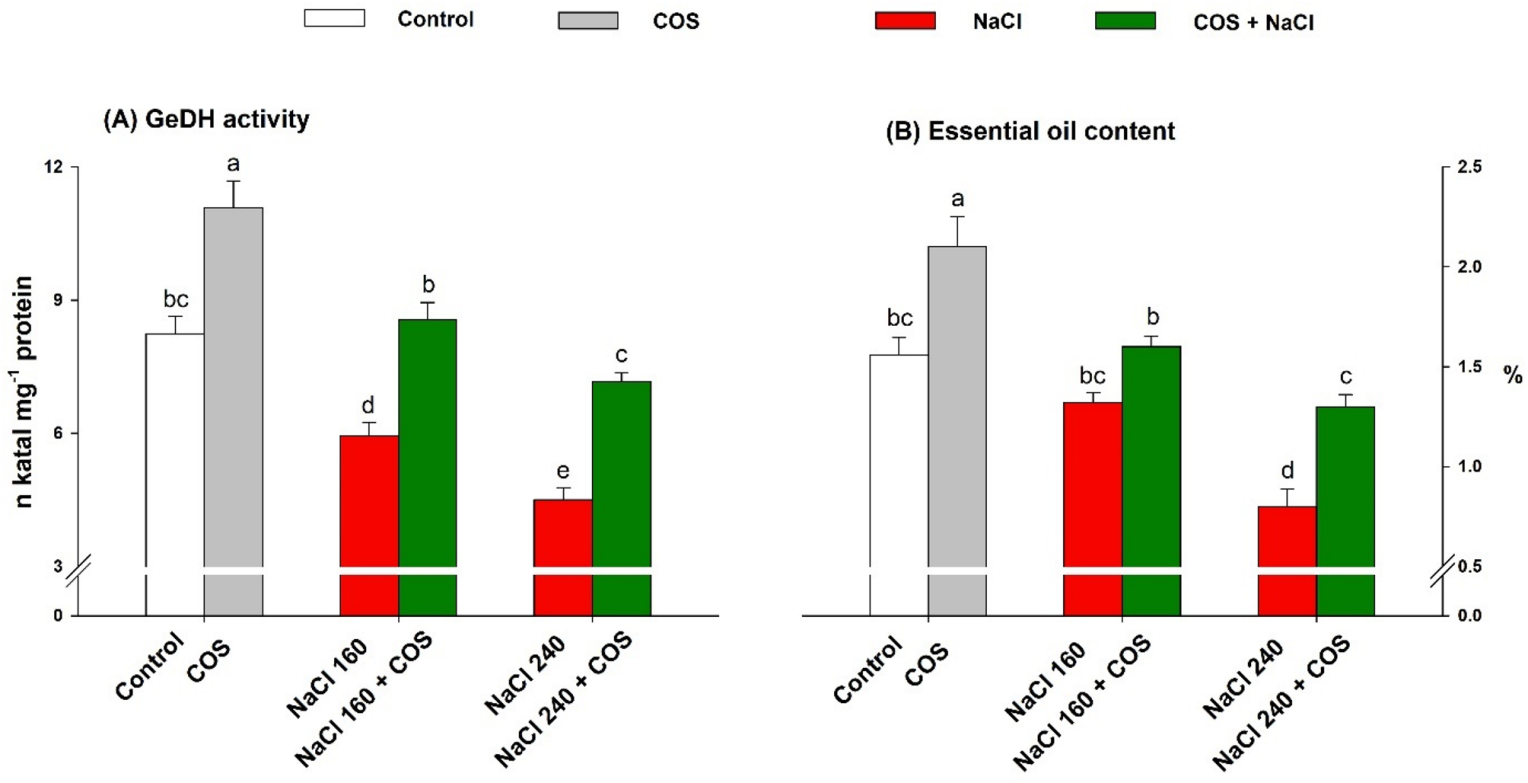
Effect of 120 mg L^−1^ chitosan oligomers (COS) sprays on geraniol dehydrogenase (GeDH) activity (**A**) and essential oil content as a percentage to plant dry weight (**B**) in lemongrass leaves during salinity stress. Five replicates mean ± standard error is represented for each bar. The difference between the mean values having the same letter(s) is insignificant (p ≤ 0.05) by the LSD test at a 5% probability level (α = 0.05). NaCl concentrations are represented in mM.

The activity of GeDH and essential oil content diminished in response to the saline treatment with the highest effect under NaCl 240 mM. GeDH activity dropped by 28% and 45% (Fig. 7A), while oil content plummeted by 15% and 49% (Fig. 7B) in NaCl 160 and 240 mM treated plants, respectively. Supplying lemongrass leaves with COS 120 mg L^−1^ redressed these cutbacks. COS application significantly raised GeDH activity in plants grown under salt conditions (NaCl 160 and 240 mM). The COS application improved essential oil content by 62.5% in plants having a soil salinity of 240 mM.

**Figure 8:**
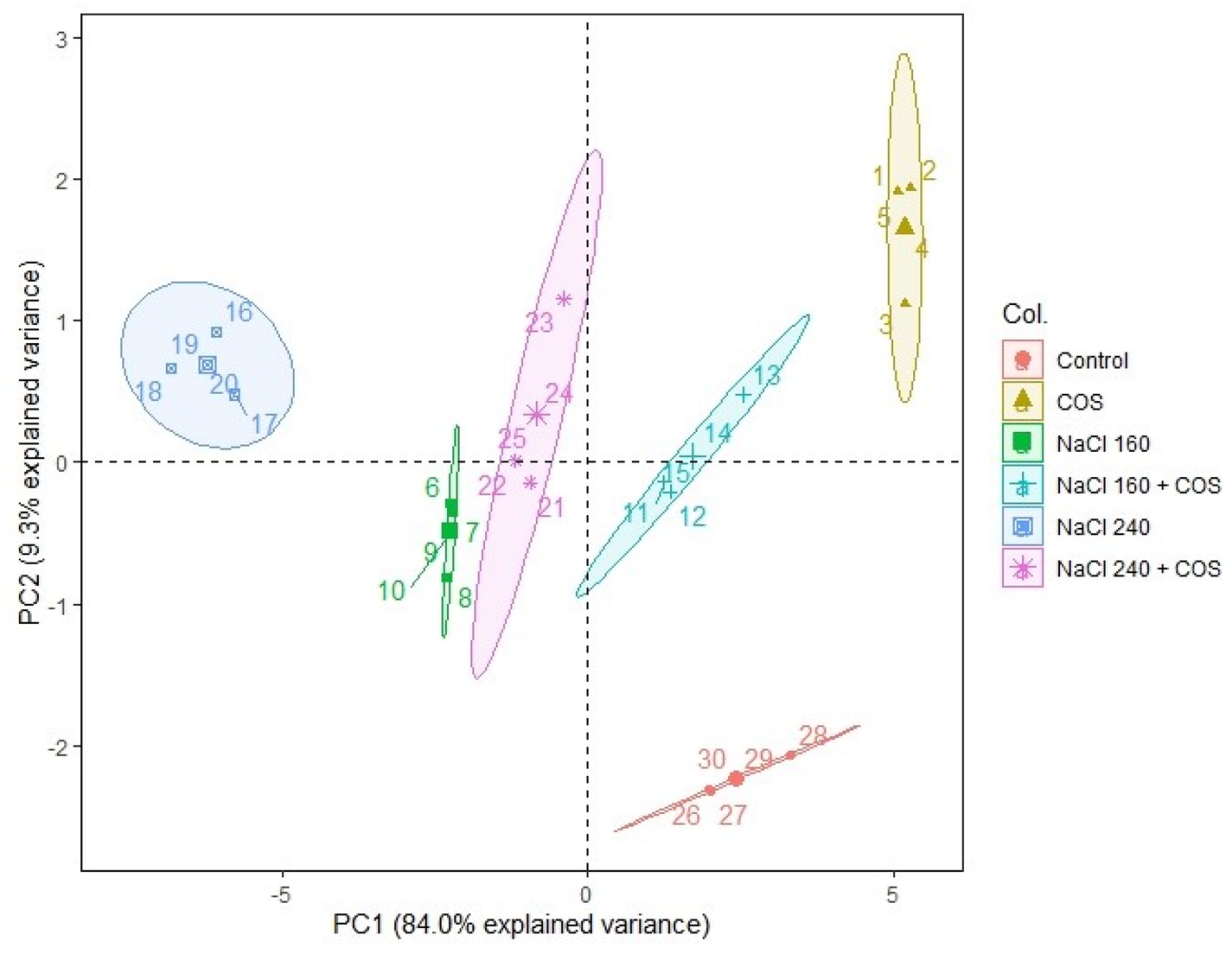
The scatter plot of PCA shows the correlation among each treatment group. The absence of overlapping clusters suggests a significant (α = 0.05) difference among treatment-induced modulations. Ellipses represent a confidence level of 95%. COS, chitosan oligomers 120 mg L^−1^; NaCl concentrations are represented in mM. The numbers illustrate the replicates of the particular treatment i.e., each treatment group consisted of 5 such replicates (n = 5).

**Figure 9:**
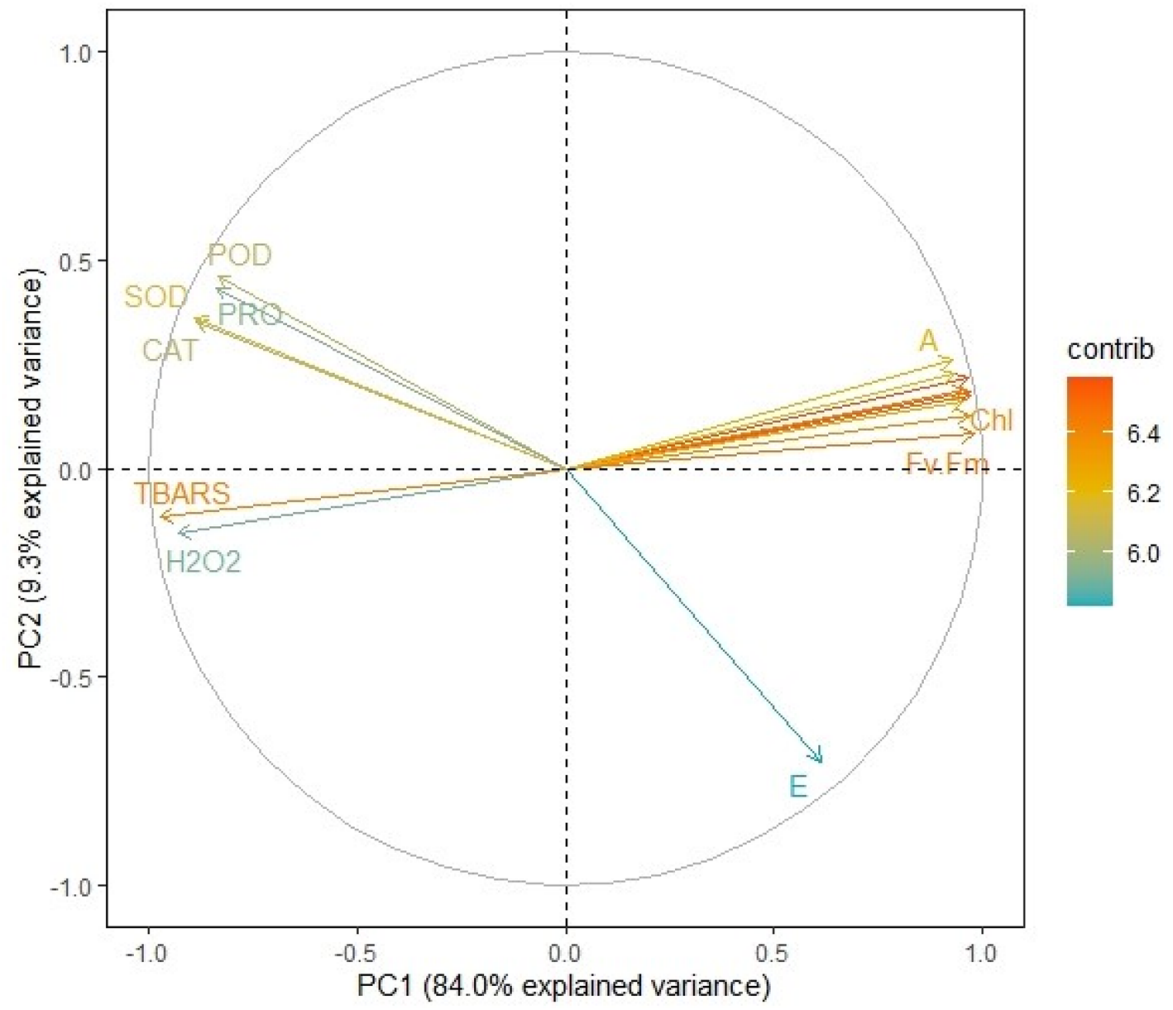
Variable correlation plot arranging the interconnection among all the variables from six treatment groups. Positively correlated variables are clubbed together while the negatively related variables are in the opposite quadrants. The distance between the variable and its origin point is directly proportional to the variables’ quality on the factor map. Colour gradients denote each variable's contribution percentage (contrib) to the principal component. *CHL* chlorophyll content, *E* transpiration rate, *A* photosynthetic CO_2_ assimilation rate, *Fv/Fm* chlorophyll fluorescence, *H*_*2*_*O*_*2*_ hydrogen peroxide content, *TBARS* thiobarbituric acid reactive substances content, *CAT* catalase activity, *POD* peroxidase activity, *SOD* superoxide dismutase activity, *PRO* proline content (overlapped variables: dry weight, plant height, leaf area, stomatal conductance, geraniol dehydrogenase activity, and essential oil content).

Principal component analysis (PCA) was performed for each studied growth, development, and productivity parameter. The scree plot analysis revealed that the first two dimensions (principal components) explain about 93% of the total variance (Supplementary Fig. 1). Therefore, the remaining components were overlooked in further PCA plots. We observed significant differences among each treatment-induced effect during the PCA scatter plot (Fig. 8). Plants treated with COS sprays held the highest explained variance with both PC1 and PC2. The same treatment also rendered maximum growth and productivity elicitations in the present study. Contrary to this, the variability of control plants and plants treated with 240 mM NaCl were least explained on PC2 and PC1, respectively. Further, the PCA variable plot shows significant correlations among variables of all six treatment groups (Fig. 9). The variables were further colour-sorted based on their contribution to the principal component. The expected average contribution for each variable to both PC1 and PC2 was 6.2% (Supplementary Fig. 2). Higher values represent a greater contribution of the variable to PC1 and PC2. The contribution of each variable to the PC1 can be found in Supplementary Fig. 3. In contrast, variable contribution to the PC2 is depicted in Supplementary Fig. 4. Moreover, we analysed how closely different parameters were related to each other among all treatments. The correlation matrix chart displayed a high correlation among various modules of growth, development, and productivity (Supplementary Fig. 5).

## Discussion

### COS recover plant growth parameters during salt stress

High saline doses (160 and 240 mM) severely damaged the growth and development of the lemongrass plants, which could be ascribed to their salt sensitiveness^58^. Higher salt concentration restricted plant height, dry weight, and leaf area. The reduced growth and development of lemongrass plants under salinity can be ascribed to osmotic and ionic imbalance, insufficient nutrient uptake, photosynthesis, and water retention in the plant^69,70^. With increasing salt concentration, plant struggles for water availability in the soil. Since salt meddles with plant mineral uptake and assimilation, the overall growth and development of the plant are reduced to a minimum^71^. Nonetheless, we observed a reversal of salinity influence on lemongrass growth and development with COS application. COS could have ameliorated salt stress by improving plant–water relation and nutrient uptake through osmotic adjustment and reducing free radical accumulation^41,72,73^. Moreover, COS could also strengthen the source-sink potential and avail more photosynthates for upregulated growth and development^74,75^. Chitosan (C_11_H_17_O_7_N_2_) has a high nitrogen content (about 7%), and it seems that nitrogen electrons could perform a pivotal role in contributing to the metal ion fixation of the chitosan. Thus, chitosan can stick with the plant longer owing to its higher chelating ability and have long-lasting effects on the plant. Further, COS may perform phytohormone-like activity altering genetic expression and manipulating cellular signalling^76^. Earlier reports have also established the eliciting activities of irradiated chitosan on the growth and development of several plants such as *Malabar spinach*^77^, *Brassica rapa*^78^, *Triticum aestivum*^79^, *Oryza sativa*^79^, *Cymbopogon flexuosus*^44^, *Glycine max*^79^, *Trigonella foenum-graecum*^80^, *Hordeum vulgare*^79^, and *Solanum tuberosum*^81^ under normal and stress environments.

### COS impact positively photosynthesis and stomatal behaviour during salt stress

Photosynthesis can be considered one of the heaviest hits under salinity stress that accounts for substantial setbacks in plant survival and productivity. Soil salinity promotes photosynthetic arrest through a wide range of stomatal and non-stomatal restrictions^82,83^. Salinity could upregulate the chlorophyllase activity, the key enzyme responsible for chlorophyll degradation; inhibit chlorophyll biosynthesis, modulate chloroplast ultrastructure through oxidative peroxidation, and influence the electron transport system^84^. The salinity retards the performance of PSII and reduces the antenna protein content by reducing the gene expression levels of these proteins, which could influence the electron transport chain and quantum efficiency of PSII^85^. The plant could also develop genetic aberrations under severe salinity, leading to downregulated photosynthetic efficiencies. These possibilities could explain the observed photosynthetic and pigmentation loss under salt stress. In addition to photosynthesis, salinity controlled stomatal behaviour substantially^82^. Our results, in line with previous studies, indicated restricted *A* and g_s_ under saline environments^86,87^. Stomatal closure could be a basic feedback mechanism to minimise the transpiration loss of the water in the lemongrass. Nevertheless, elongated stomatal closure during salinity reduces CO_2_ intake and, subsequently, carbon assimilation, plummeting the net CO_2_ assimilation rate and resulting in carbon starvation^88^. However, we observed an outright opposite pattern in such phenomena with COS supplementations. COS treatments promoted chlorophyll content, photosynthetic efficiencies, and stomatal physiology in lemongrass plants. COS upregulated g_s_ under salinity, boosting CO_2_ assimilation that might have overcome salinity-induced carbon starvation in lemongrass. Interestingly, unstressed plants treated with COS show increased g_s_ while the transpiration rate decreases. One hypothesis could be considering chitosan’s capability to hold water molecules to maintain a higher plant-water status. Thus, although more stomata were open, relatively lesser water molecules transpired. However, we do not have enough data at this point to strongly support this hypothesis. Nevertheless, the COS treatments improved gas-exchange parameters under salt regimes which denotes the beneficial effect of COS under salinity stress. Various studies have reported that COS could directly influence chlorophyll biosynthesis and thus influence photosynthetic efficiency and productivity^41,89^. Reduced photon loss as heat dissipation with COS sprays and improved electron transport rate could have assisted in the ultimate photosynthetic and stomatal improvement in the present study. Others developed similar understandings of COS action mechanism in different crops such as *Zea mays*^90^, *Solanum tuberosum*^81^, *Hordeum vulgare*^79^, *Triticum aestivum*^38^, and *Brassica rapa*^78^.

### COS-induced cellular antioxidant defence during oxidative stress induced by salinity

The key ROS are H_2_O_2_, superoxide anion (O_2_^**·**−^), singlet oxygen (^1^O_2_), and hydrogen radical (^**·**^OH) which are produced primarily in the electron transport chain during chloroplastic photosynthesis, mitochondrial respiration, peroxisomes (photorespiration and β-oxidation), plasma membrane-bound respiratory burst oxidase homologue (RBOH), as well as other components present in the vacuole, endoplasmic reticulum, cytoplasm, and apoplast^8,10,11,91,92^. Salinity triggers ROS production which prompts cellular damage by destabilising proteins, membrane lipids, and nucleic acids and builds up oxidative stress^1,70^. We observed similar oxidative bursts in terms of increased TBARS and H_2_O_2_ content in salinity-exposed lemongrass plants.

However, plants treated with chitosan nanoparticles could minimise salinity-conferred lipid peroxidation and membrane permeability change through boosted antioxidants and alkaloid biosynthesis in *Catharanthus roseus*^73^. The COS-supplied lemongrass had increased SOD, CAT, and POD activities, as well as the PRO content. SOD reduces O_2_^**·**−^ to less reactive H_2_O_2_ molecules and is considered the first line of enzymatic defence against oxidative damage^93,94^. This H_2_O_2_ influx is controlled by CAT and POD reducing it to stable water molecules. While salinity is attributed to increasing the O_2_^**·**−^ and H_2_O_2_ content, COS has been reported to upregulate the activities of SOD, CAT, and POD^34,44^. COS might have upregulated the expression of various defence-related genes to maintain redox homeostasis^95–97^. Chitosan and its derivatives support the antioxidative system in several crops during salinity with their antioxidant and radical scavenging affinity^98–102^. The positive role of COS on osmoprotection in lemongrass can be observed by increased PRO content since PRO is an efficient osmolyte against salinity-induced osmotic stress^103^.

### COS upregulate essential oil biosynthesis during salt stress

Essential oil productivity in lemongrass is a highly regulated process and can be influenced by several factors including extraction method, plant developmental stage, and environmental conditions^58,104^. The plummet in LEO content under salinity could result from poor plant growth and development owing to ionic, osmotic, and oxidative imbalance, and retarded plant-water relation, nutrient uptake, photosynthates production, and source-sink potential^51,105,106^. Nevertheless, COS upregulated essential oil productivity in lemongrass under both saline regimes i.e., NaCl 160 and 240 mM. GeDH enzyme also exhibited enhanced activity under these scenarios. COS application seems to support stomatal behaviour, photosynthesis, cellular homeostasis, and several enzyme activities including GeDH^41,73^. Since chitosan and its derivatives have phytohormone-like behaviour and can act as signalling molecules, increased GeDH activity in the present study may have resulted from COS-induced expression of transcripts responsible for GeDH biosynthesis^81,107^.

**Figure 10:**
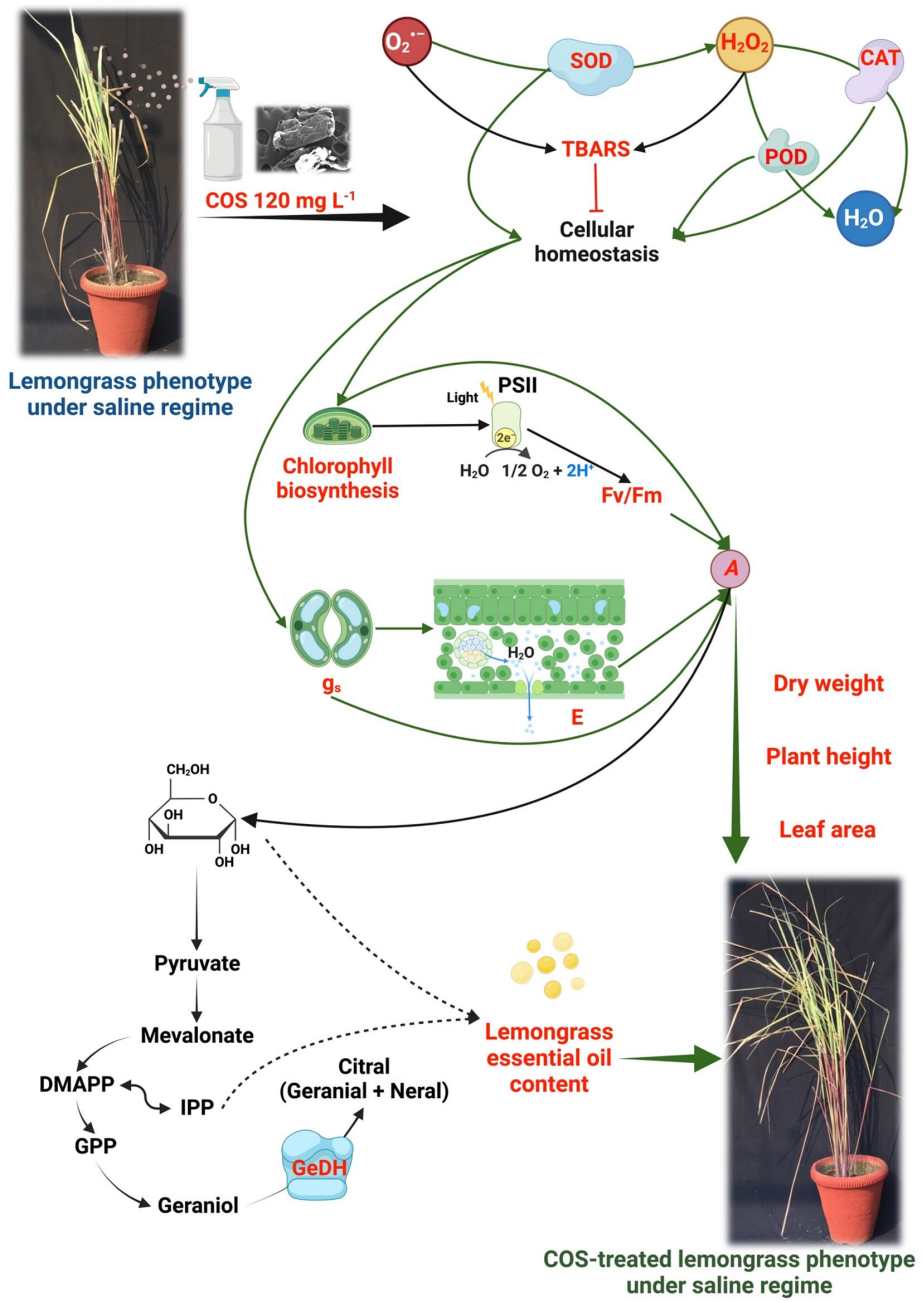
Proposed *modus operandi* of chitosan oligomers (COS) in lemongrass as was developed during the present study. Our results suggest that COS palliates salt-induced oxidative stress by boosting antioxidant metabolism (such as SOD, CAT, and POD). Improved cellular homeostasis could support chlorophyll biosynthesis and PSII efficiency (Fv/Fm). Subsequent upgradation in stomatal dynamics (such as g_s_ and *E*) would assist lemongrass with a higher photosynthetic CO_2_ assimilation rate (*A*). Further, a higher *A* is expected to generate more glucose which can undergo a mevalonate or mevalonate-independent pathway to confer improved essential oil productivity in salt-stressed lemongrass. The overall upgradation of plant physiology during salt stress can render morphological improvements in lemongrass such as dry weight, leaf area, and plant height. The studied phenomena are coloured in red while the green arrows show COS-induced elicitation of the process.

In summary, our results indicate that COS application upgrades plant physiology and triggers enhanced cellular defence in lemongrass against high salinity. COS-assisted Fv/Fm and g_s_ during saline conditions promise improved plant growth and development. Further, lemongrass plants were better prepared for salinity with COS on cellular levels since they showed an upregulated ROS and antioxidant metabolism over control plants. The intensified SOD, CAT, and POD activities work to maintain cellular homeostasis. These, in concert, brought higher crop productivity in the present study. Therefore, it is proposed that COS could be a useful biotechnological tool to palliate salinity-induced oxidative stress in lemongrass crops and that its use could be extrapolated to other agricultural species. A working model for these coordinated biochemical effects is proposed in Fig. 10 which is based on our understanding developed during the present study and the insights from our previous studies with lemongrass (see reference list for details).

## Supplementary Information

Supplementary Information.

## Data Availability

All data supporting the findings of this study are available within the paper.
