# Supplementary material for: Chitosan oligomers (COS) trigger a coordinated biochemical response of lemongrass (Cymbopogon flexuosus) plants to palliate salinity-induced oxidative stress
Source: Sci Rep. 2023 May 27;13:8636. doi: 10.1038/s41598-023-35931-w (PMC10224966; doi:10.1038/s41598-023-35931-w)
Supplement: Supplementary file 1 — Supplementary Information. [file 41598_2023_35931_MOESM1_ESM.docx]

**Chitosan oligomers (COS) trigger a coordinated biochemical response of lemongrass (*Cymbopogon flexuosus*) plants to palliate salinity-induced oxidative stress**

Mohammad Mukarram^1,2,*^, M. Masroor A. Khan^1^, Daniel Kurjak^3^, Francisco J Corpas^4^

^1^Advance Plant Physiology Section, Department of Botany, Aligarh Muslim University, Aligarh 202002, India

^2^Department of Phytology, Faculty of Forestry, Technical University in Zvolen, T. G. Masaryka 24, 96001 Zvolen, Slovakia

^3^Department of Integrated Forest and Landscape Protection, Faculty of Forestry, Technical University in Zvolen, T. G. Masaryka 24, 96001 Zvolen, Slovakia

^4^Department of Stress, Development and Signaling in Plants, Group of Antioxidant, Free Radical and Nitric Oxide in Biotechnology, Food and Agriculture, Estación Experimental del Zaidín, Consejo Superior de Investigaciones Científicas (CSIC), Granada, Spain

***Correspondence:** Mohammad Mukarram ([mdmukarram007@gmail.com](mailto:mdmukarram007@gmail.com))

**ORCID:** Mohammad Mukarram (0000-0002-9034-9366), M. Masroor A. Khan (0000-0002-4530-9082), Daniel Kurjak (0000-0002-2489-8463), Francisco J Corpas (0000-0002-1814-9212)

**Running title:** Chitosan oligomers boost plant tolerance to salt stress.


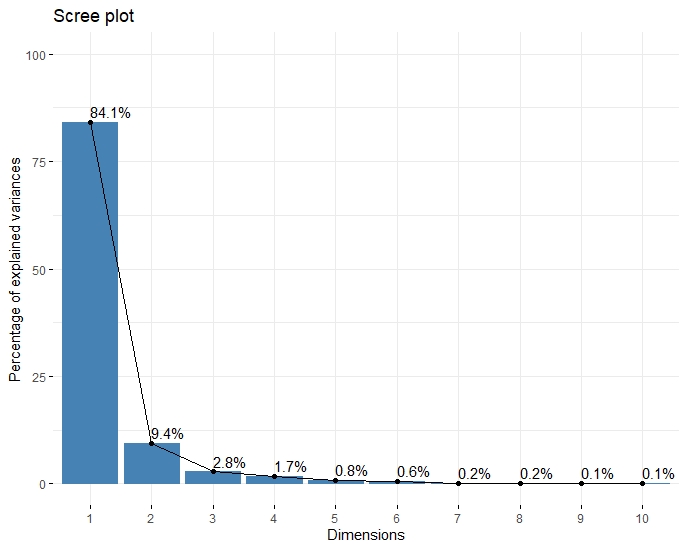


**Supplementary Fig. 1** Scree plot displaying first two principal components (PC1 and PC2) capturing the most variation i.e., about 93%.


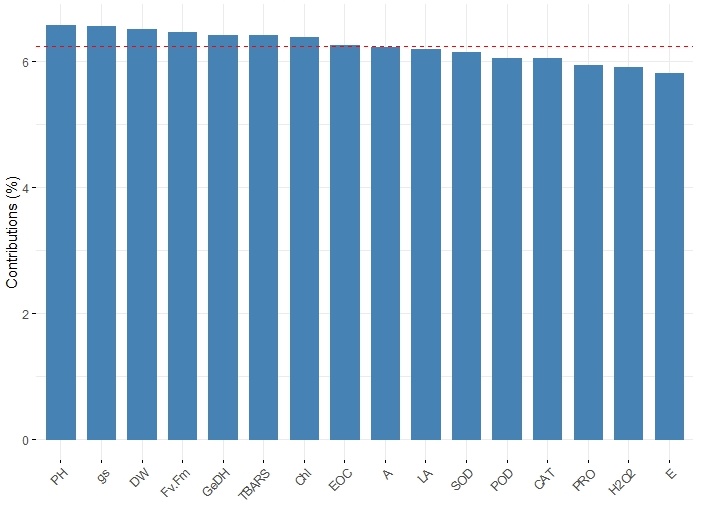


**Supplementary Fig. 2** Contribution percentage of each variable to both principal components
i.e., PC1 and PC2 where the first seven variables exhibited higher contribution percentages than the average (6.2%).). The broken red line points to the expected average contribution percentage of each variable to PC1 and PC2. PH, plant height; gs, stomatal conductance; DW, dry weight; Fv.Fm, chlorophyll fluorescence; GeDH, geraniol dehydrogenase activity; TBARS, thiobarbituric acid reactive substances content; Chl, chlorophyll content; EOC, essential oil content; A, photosynthetic CO_2_ assimilation rate; LA, leaf area; SOD, superoxide dismutase activity; POD, peroxidase activity; CAT, catalase activity; PRO, proline content; H2O2, hydrogen peroxide content; E, transpiration rate.


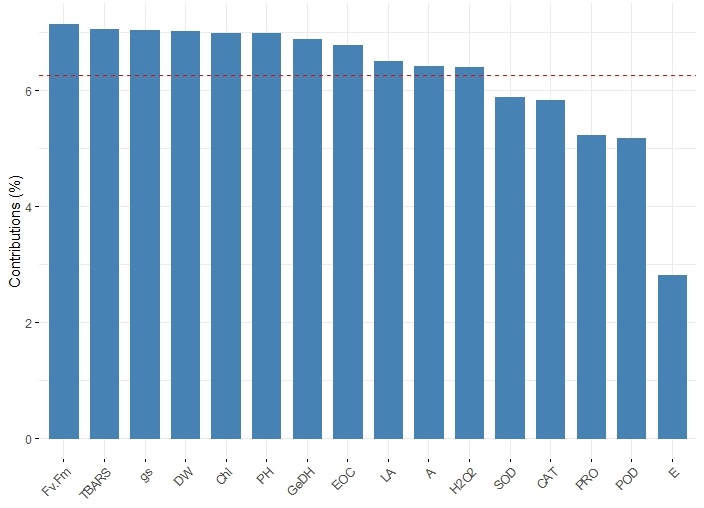


**Supplementary Fig. 3** Contribution percentage of each variable to first principal component (PC1). The broken red line points to the expected average contribution percentage of each variable to the PC1. Fv.Fm, chlorophyll fluorescence; TBARS, thiobarbituric acid reactive substances content; gs, stomatal conductance; DW, dry weight; Chl, chlorophyll content; PH, plant height; GeDH, geraniol dehydrogenase activity; EOC, essential oil content; LA, leaf area; A, photosynthetic CO_2_ assimilation rate; H2O2, hydrogen peroxide content; SOD, superoxide dismutase activity; CAT, catalase activity; PRO, proline content; POD, peroxidase activity; E, transpiration rate.


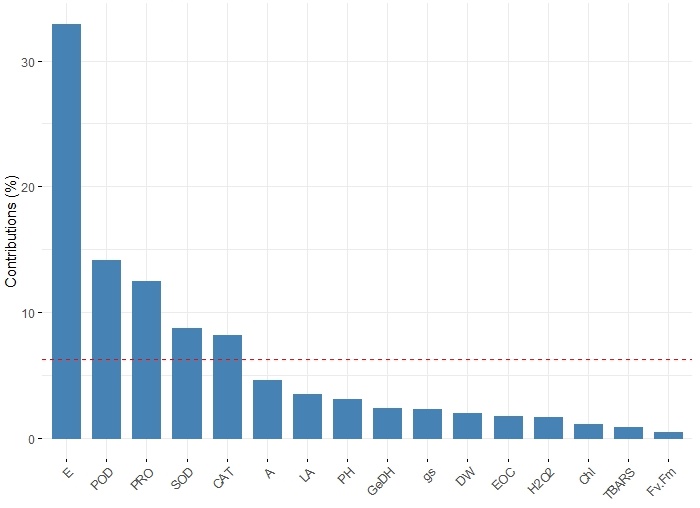


**Supplementary Fig. 4** Contribution percentage of each variable to second principal component (PC2). The broken red line points to the expected average contribution percentage of each variable to the PC2. Contrary to PC1, transpiration rate (E) contributed the highest percentage to the PC2. E, transpiration rate; POD, peroxidase activity; PRO, proline content; SOD, superoxide dismutase activity; CAT, catalase activity; A, photosynthetic CO_2_ assimilation rate; LA, leaf area; PH, plant height; GeDH, geraniol dehydrogenase activity; gs, stomatal conductance; DW, dry weight; EOC, essential oil content; H2O2, hydrogen peroxide content; Chl, chlorophyll content; TBARS, thiobarbituric acid reactive substances content; Fv.Fm, chlorophyll fluorescence;

**
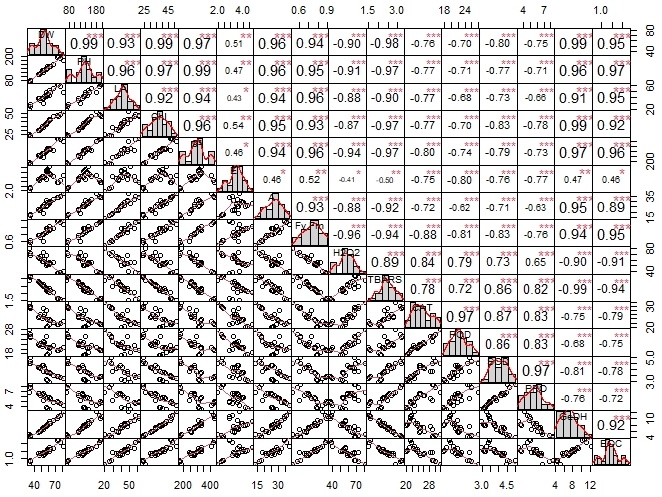
**

**Supplementary Fig. 5** Chart of the correlation matrix exploring the intricate relation among variables using Pearson’s method. Each variable is distributed on the chart diagonal. The bottom of the diagonal corresponds to the bivariate scatter plot with a fitted line between any two variables. Further, each box in the diagonal top consists of the correlation values between any two variables along with significance levels. Significance levels are denoted by asterisks following p-values. (p=0.01), * (p=0.05), ** (p=0.01), *** (p=0.001). DW, dry weight; PH, plant height; LA, leaf area; CHL, chlorophyll content; gs, stomatal conductance; E, transpiration rate; A, photosynthetic CO_2_ assimilation rate; Fv/Fm, chlorophyll fluorescence; H2O2, hydrogen peroxide content; TBARS, thiobarbituric acid reactive substances content; CAT, catalase activity; POD, peroxidase activity; SOD, superoxide dismutase activity; PRO, proline content; GeDH, geraniol dehydrogenase activity; EOC, essential oil content.
